# Achieving Value-Based Care in Chronic Disease Management: Intervention Study

**DOI:** 10.2196/10368

**Published:** 2019-05-03

**Authors:** Nilmini Wickramasinghe, Blooma John, Joey George, Doug Vogel

**Affiliations:** 1 Swinburne University of Technology Hawthorn Australia; 2 Epworth HealthCare Richmond Australia; 3 Peter MaCallum Cancer Centre Melbourne Australia; 4 University of Canberra Canberra Australia; 5 Iowa State University Ames, IA United States; 6 Harbin Institute of Technology Harbin China

**Keywords:** diabetes, gestational diabetes, chronic disease management, value-based care, mobile health, power knowledge, Australian health care system, 2-period 2-arm crossover, clinical trial

## Abstract

**Background:**

The World Health Organization notes that diabetes, a chronic disease, is a silent epidemic, and by 2020 there will be a 54% rise in the total number of individuals diagnosed with this disease. These are alarming figures that have significant repercussions for the quality of life of individuals and their families as well as for the financial stress of health care systems globally. Early detection and proactive management of diabetes is essential. The Diamond solution provides diabetes self-management by enabling patients to send details about their blood sugar readings at specific times to their nominated care coordinator to receive recommendations for diet and exercise and insulin titration.

**Objective:**

The aim of the study was to assess the usability, acceptability, and fidelity of the Diamond diabetes monitoring device for patients with gestational diabetes mellitus (GDM). Specifically assessed were (1) patient compliance, (2) patient satisfaction, (3) level of glycemic control achieved, and (4) health professional satisfaction.

**Methods:**

Using a design science research perspective, the Diamond diabetes monitoring device solution was adapted to the Australian health care environment. Once the solution was deemed fit for purpose by the director of the OB/GYN clinical institute and on securing all relevant ethics approvals, a 2-period 2-arm nonblinded crossover clinical trial was conducted for 8 weeks total time with crossover at 4 weeks to establish proof of concept, usability, and fidelity. The patient perspective was assessed by using structured questionnaires at 4 specific stages of the project, while the clinician perspective was captured via semistructured interviews and unstructured questionnaires.

**Results:**

The 10 patients studied reported preferring standard care with the technology solution to standard care alone. Further, all clinicians involved concurred that the technology solution greatly assisted their ability to provide higher value patient-centered care. They also noted that it was extremely helpful for assisting in systematically monitoring glucose levels and any/all changes and trends.

**Conclusions:**

Based on these initial findings, we offer a holistic pervasive approach to enable the achievement of value-based, patient-centered care in chronic disease management. Key lessons include the importance when designing such solutions to focus on the two primary user groups (patients and clinicians).

## Introduction

Diabetes mellitus is one of the leading chronic diseases, and its prevalence continues to rise exponentially. The number of diabetes patients worldwide is estimated to rise to 366 million in 2030 from 171 million in 2000 [1]. Australia is expected to be a significant contributor to this projected trend. As of June 2016, more than 1.2 million Australians had been diagnosed with diabetes and were registered with the National Diabetes Services Scheme [2]. This number represents those diagnosed with type 1, type 2, and gestational diabetes.

An estimated 280 Australians develop diabetes every day [2,3]. Moreover, for every person diagnosed with diabetes, it is estimated that there is another who has yet to be diagnosed, which doubles the number of people with diabetes. If uncontrolled or poorly managed, diabetes can lead to chronic vascular and kidney diseases, strokes, heart attacks, eye diseases, and neuropathy and, for some individuals, amputations of extremities and limbs [4,5]. Furthermore, diabetes and its complications incur significant costs for the health system in Australia, including costs incurred to careers, government, and the entire health system.

Treatment of women with gestational diabetes mellitus (GDM) aims to control maternal, and therefore fetal, hyperglycemia and the associated tendency of fetal hyperinsulinemia, which is at the root of fetal complications [6]. After many years of uncertainty as to the value of such treatment in GDM, two key trials have now shown benefit for both mother and fetus for antenatal initiation of lifestyle modification and glucose monitoring coupled with insulin therapy as necessary [7,8]. Antenatal treatment of detected mild GDM was also associated with improved health status for women during the antenatal period and at 3 months after birth, with less postnatal depression [8]. Specifically, there is agreement in the literature that specific self-management activities including glucose monitoring, dietary restrictions, and exercise regimes can result in good outcomes for mothers and babies, suggesting that self-management behaviors can be critical [7,9]. More recently in Australia, there has been a lowering of the threshold level for when a pregnant woman is classified as having GDM, which immediately led to a significant increase in the number of women diagnosed with GDM over and above the growing trend that has been occurring. This change in classification makes it even more pressing to find a suitable solution.

Early detection and proactive management of diabetes is essential [10,11]. A critical treatment imperative is to provide patients with diabetes appropriate monitoring to enable better assessment and control of blood glucose and prevent further complications [12]. It is also essential that a cost-effective solution convenient to patients and clinicians and least disruptive to the patient lifestyle be adopted [13].

Inet International developed a pervasive technology solution to facilitate patient empowerment with their care [14-17]. The solution uses pervasive mobile technology to transfer critical information between patient and providers, ensuing in superior monitoring. This solution has proved successful in assisting to lower hemoglobin A_1c_ (the universally recognized marker for diabetes) in trials in Canada and the United States [12].

We report on the findings of our clinical study, based on our holistic pervasive approach, to enable the achievement of value-based care in chronic disease management that is patient-centric and focuses on the two primary user groups (patients and clinicians). Given that health care costs are an important aspect of all health care agendas today, we frame our recommendations against a value-based paradigm, as we believe this a responsible approach to take. Moreover, a key emergent aspect of the study was the power-knowledge dynamic that exists between patients and their clinical care team. We expand on this finding, noting how it might influence adoption and use of the technology solution. In particular, we note that when developing technology solutions, it is important to engage both user groups; without clinician support and engagement, it is unlikely that patients will be willing to adopt or use a technology solution.

The aim of this study was to assess the usability, acceptability, and fidelity of the pervasive technology solution (Diamond, a diabetes monitoring device) for patients with GDM and thereby establish proof of concept. Specifically, the study was designed to assess (1) patient compliance, (2) patient satisfaction, (3) level of glycemic control achieved, and (4) health professional satisfaction. From this, we expected it would be possible to develop a deeper understanding of the benefits, barriers, and facilitators as well as any possible negative impacts of such pervasive mobile solutions in supporting and enabling superior chronic disease management. In addition, the study served to answer the following research questions:

- How does a mobile solution enable and support the value-based care paradigm in the context of chronic disease management?
- What are the benefits and suitability of such a pervasive technology solution to self-care?
- What are the key barriers and facilitators for the application of a pervasive technology solution to support GDM patient care?
- What are the possibilities of applying the tools and techniques of data science to enable precision health care delivery and inform public health care initiatives regarding better chronic disease management practices and protocols?
- Are patients influenced and persuaded by their clinician to adopt the solution and is this important in choosing the solution?

## Methods

### Study Site

The data site chosen to conduct our study, a large tertiary, not-for-profit health care system, is situated in Melbourne, Australia. The Australian health care system is essentially a two-tier complementary system [18]. This means that all citizens and permanent residents have basic health care provided by a national government scheme called Medicare and can choose to take on additional coverage via private health care insurance. In Australia, the health care system has historically been centered on the practitioners and service providers. It is a highly fragmented system with both state and federal government jurisdiction. Hence, there exist many types of health care providers from solo practice to public hospitals (government hospitals) to various types of private hospitals. The chosen data site is in the private system; hence, patients receiving treatment at this hospital must have private insurance. In addition, the hospitals in this system are tertiary, which means they conduct leading research to strive to discover better ways to treat medical issues, and their not-for-profit status means that any surplus is reinvested into the system.

### Diamond Diabetes Monitoring Device

The Diamond solution was developed by the Canadian company Inet International to provide diabetes self-management and monitoring to all patients diagnosed with diabetes. Key aspects of the solution include full compliance with the US Health Insurance Portability and Accountability Act; it is totally pervasive in that it works on any mobile platform (Android, iOS, etc) and it requires co-use or coadoption by the patient and their clinical care team.

Diabetic patients provided with the Diamond solution are able to send details about their blood sugar readings at specific times (ie, before breakfast, 2 hours after breakfast, 2 hours after lunch, and 2 hours after dinner) to their nominated care coordinator (typically a diabetic educator or endocrinologist or other doctor). On receiving this information, the care coordinator will respond to the patient with recommendations for diet and exercise and insulin titration. This exchange happens in real time. In addition, the solution keeps a log of diet and exercise activity and insulin use and graphs the blood sugar readings over time so that both patient and care coordinator can observe what is happening at any time.

### Ethics

The Australian research code of ethics classifies any clinical intervention on pregnant women at the highest risk and, hence, the ethics process is at a national approval level and very strict. Ethics approval was received from the Epworth Human Research Ethics Committee, but the ethics committee limited the sample to 10 patients given that the clinical study was the first of its kind to be administered on pregnant women, and thus, fell into a high-risk category; if a patient dropped out, for whatever reason, we were able to add another patient.

To tailor the chosen pervasive technology solution, Diamond, to the specific health care context of the data site as well as comply with legal and ethical requirements for use of a technology solution in a study with pregnant women, it was necessary to make several tweaks to the technology solution so that it was both compliant and fit for purpose. We complied with all requirements and secured all necessary ethics clearances; to do this in a systematic fashion, we employed a design science research methodology approach (Multimedia Appendix 1).

### Research Design

**Figure 1:**
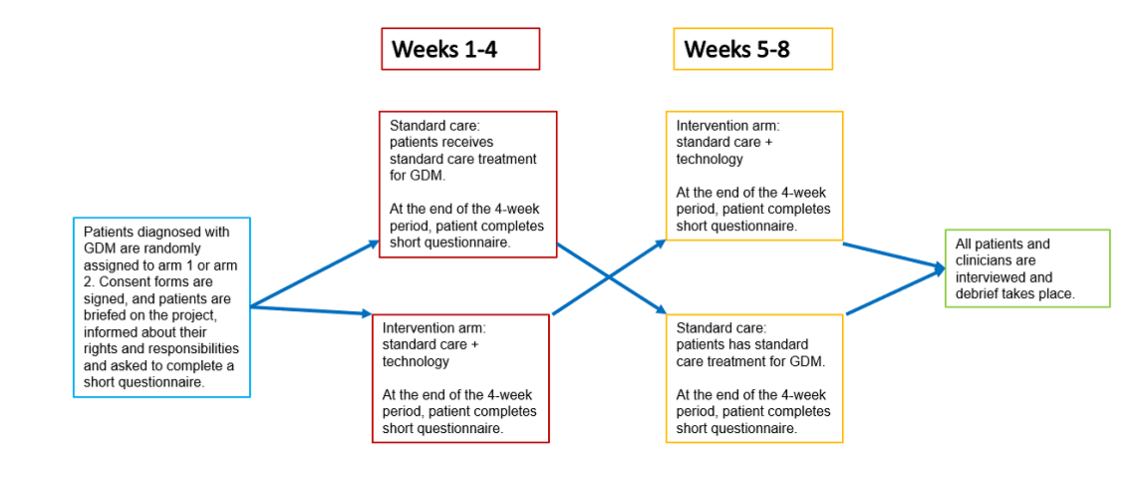
Study design. GDM: gestational diabetes mellitus.

A 2-period, 2-arm crossover clinical trial strategy [19] with an 8-week duration per patient was adopted with two equal periods of 4 weeks each (Figure 1). As we wanted to understand the benefits, if any, of using a technology solution in addition to standard care protocols, it was necessary to have 2 arms to the crossover study design so that all patients experienced standard care and standard care plus technology. One arm was the control (ie, standard treatment of GDM by the hospital) and the other arm was the intervention (ie, standard care plus technology). In compliance with ethics regulations and laws in Australia, no patient can be denied standard care, so the intervention arm was designed as standard care plus the technology solution.

Patients were offered the opportunity to participate in the trial once a diagnosis of GDM had been made based on a glucose tolerance test administered between 26 and 28 weeks of pregnancy. Enrollment in the study was done by the endocrinologist under the supervision of the consultant obstetricians and was totally optional. At the time of enrollment, all pertinent information regarding the study was shared with patients including the crossover strategy employed. Patients were asked to complete a short questionnaire exploring demographic details, their familiarity with technology in general, and their understanding of GDM. Following enrollment in the study, patients were randomly allocated to either the standard care or standard care plus technology arm of the study. All patients were then educated in the technique of blood glucose monitoring by a diabetic educator as per standard clinical practice. They were also educated in the use of traditional or technology-based recording techniques for blood glucose.

On the advice of the obstetrics and gynecology professionals, the duration was set at 8 weeks. This was deemed suitable as it was unlikely a baby would be delivered during this time frame and we would have time for the patient to experience both arms of the study. Hence, crossover was set at 4 weeks. As noted by Rigby [19], it is ideal to have a crossover time so that all participants can experience treatment with and without the technology and then comment on the differences. It is also recommended to have participants start with the technology solution and cross to without and vice versa so that it is possible to identify any biases more easily with technology use. The patient perspective was assessed at four specific stages of the project (Multimedia Appendix 2, part A):

- Structured questionnaire at the start of the project
- Structured questionnaire at the end of using standard care
- Structured questionnaire at the end of using the technology solution in conjunction with standard care
- Structured questionnaire at the end of the project

We note that to comply with ethics requirements, we did not interview patients at the completion of the study as it was considered too much stress and imposition on a new mother.

As there were two key user groups, patients and clinicians, it was necessary to also understand findings from the clinical team. The clinical care perspective included a focus on three key members of the patient care team: obstetrician, endocrinologist, and diabetes educator. These individuals were presented unstructured questionnaires to complete at the start and end of the study and were also invited to an interview where they were asked unstructured and semistructured questions about the study and their opinions on the role and benefit of the technology solution (Multimedia Appendix 2, parts B and C).

Clinicians participated in both arms of the study and were asked about the crossover aspects during their interview at the end of the study. It was our desire to have questionnaires for the clinicians at the point of crossover, but ethics reviewers stated that this was superfluous and clinicians should not be given more questionnaires than necessary, so they were removed to comply with ethics requirements. The objective of the questionnaire at the start of the study was to assess ease of use with technology in day-to-day life determined by mobile solution used and frequency of use. The questionnaire at the conclusion of the study was designed to ascertain overall satisfaction with the technology solution and allow for any recommendations moving forward.

## Results

To address the stated aims and answer the research questions, this study analyzed the data from two key perspectives: patient and clinician. Subscribing to the directives of Boyazis [20], the qualitative data were analyzed by examining the occurrence and frequency of the a priori themes and then any emergent themes. Qualitative and qualitative data were collected from the surveys conducted for both patients and clinicians as well as from performing basic statistical analysis on the numbers of patients in the study.

From the patient perspective, the key a priori themes included: (1) health literacy and understanding regarding diabetes management, (2) general familiarity with and use of mobile solutions, (3) standard care, (4) technology solution, and (5) suggestions to enhance the technology solution. Further, thematic analysis served to uncover other results from the patient data collected: (1) their supportive care team played a big role in their being comfortable with the technology solution because they trusted their doctor and were confident with the doctor’s decision and (2) they were committed to doing whatever was best for their unborn baby’s health, so having timely advice made them feel they were doing the best for their baby.

Overall, a wonderful initiative. This app makes it easier for patients with GDM as it’s quick to enter readings, easy to track trends using graph on website and an effective and efficient way to communicate with doctors/nutritionists. For busy mums-to-be especially this is a fantastic tool, easier than remembering to call with readings each week. Excellent concept!

Patient 02

I could do it [record and check blood glucose levels] any time of day that was suitable and convenient to me. Knowing that someone was on the other end and would contact me if there was a concern/further information required. Info would be in writing. Could maintain privacy at work, no need to duck out to have conversations and report BGL [blood glucose level]. Didn’t need pen and paper.

Patient 07

In addition, all patients preferred to have standard care with the mobile solution rather than the standard care approach only. All patients used mobile phones daily and felt very comfortable entering the required data. They all had a good understanding of the protocols they should follow for GDM once they were explained by their health care professional, and they complied as best as they could. This is not uncommon, given most mothers-to-be try to do what is in their unborn baby’s best interests. At the conclusion of the study, many ideas for further enhancement were provided by the patients, including having a recommended food diary, assistance on where to get the needed food, recommendations for alternate exercise, and voice recognition to avoid data entry. All patients completed the four questionnaires.

For consistency, the same a priori themes were used to examine data collected from the clinicians. Overall, all the clinicians preferred the technology solution over the standard care only scenario. A total of 60% (6/10) of clinicians were totally happy using the app, and it was acceptable “as is” for them, while 40% (4/10) were happy with the solution but thought it could be further enhanced and was only useful for typical GDM patients. They had reservations about using the solution in the case of complicated GDM patients. We note that, given ethics clearances obtained, we could not include high-risk patients in the study, so we cannot show any results for high-risk patients. Enhancements included keeping track of weight and blood pressure on an ongoing basis and graphing these together with the blood glucose levels.

Further, the clinicians concurred that the technology solution greatly assisted their ability to save time and made the monitoring of gestational diabetes more efficient and effective and was extremely helpful to assist in systematically monitoring glucose levels, changes, and trends any time regularly. This enabled them to provide better/optimal care for their patients; as they noted, having the key data at the right time greatly assisted them in making prudent decisions. They noted that even 1- to 2-point differences in blood glucose in pregnancy can have a severe impact on the fetus as it develops, given the resulting relatively much higher concentration of fetal blood glucose. For a tiny fetus, every extra second of exposure to a higher concentration of blood glucose can lead to major issues at birth and even throughout life.

In terms of usability, there were suggestions to change the format of patient entry so that it was clear when blood levels were seriously out of range versus slightly out of range. The only concern from the diabetes educators pertained to communicating with patients through the app rather than face-to-face appointments when dealing with a large sample size and the likely time requirements this would involve. It is anticipated that with appropriate staffing and workflow processes in place, this should not be an added burden, and it will also lend itself to capturing large amounts of data in a systematic fashion. These data can then be analyzed, so potential benefits at a more population health–level might ensue, which in turn might lead to new insights into diabetes management, treatment, and even prevention.

The clinicians in the study were particularly interested to further investigate the potential of the data and how data analytics might be used to identify key trends. They noted that GDM is the least understood form of diabetes and having cohorts of data on GDM patients would provide immense value in assisting to better understand this disease. They believe that the data collected could be helpful to examine what diets and exercise and when these occurred was best with GDM management as well as other factors including ethnicity and age. To get such a rich picture and understanding would assist them to develop better protocols for their patients and even contribute to public health protocols.

The clinicians identified as a major barrier hospital regulations and legal and government aspects.

In obstetrics there is always much focus if things go wrong—it is not good for the hospital, and the government and legal issues are complex, and it tends to get quite emotional too. Thus it tends to be quite conservative. Without hospital executive support, it is not possible to move forward with technology solutions. This is key, especially in our area.

OBGYN 01

In addition, emergent themes developed that focused on the need for coaching and education, need to redesign existing operations to make the best use of the efficiency and effectiveness potential the technology solution affords, and concerns about time commitments required and managing expectations regarding response rates by a member of the clinical care team. Finally, clinicians identified that it would be good to further enhance the solution to provide monitoring and management postdelivery to ensure that the mother still controls her blood glucose levels.

## Discussion

### Principal Findings

On analysis of the collected data, we contend that acceptability, usability, and fidelity were established as was initial proof of concept of the solution. Specifically, all patients using the technology solution with standard care had better management of blood glucose levels and were able to monitor and manage their GDM together with their clinician more effectively and efficiently when compared with standard care only. This was based on daily readings, examination of medical records and reports, and patient and clinician feedback. We note that the sample size was small (and this was due to ethics restrictions as already mentioned); however, we believe that by running further confirmatory studies we can develop a larger evidence base to further demonstrate the benefits of the Diamond solution for supporting and enabling superior care for patients with GDM. Moreover, the study establishes the benefits of such mobile solutions to both patients and their clinical care team to be used as an adjunct with standard care protocols. Specific answers to each of the posed research questions are provided in Multimedia Appendix 3. We note that while the results obtained may only be pertinent in the context of GDM, the developed framework can be generalized to other types of diabetes and even other types of chronic care interventions that use mobile solutions. We plan to expand on this in our future work.

### Limitations

Like all studies, this study had a few limitations Sample size was limited due to ethics requirement. This in turn means that with such a small sample size generalizability is not possible. However, this directional data gives insights as to what might be appropriate in other contexts, which our follow-up research will investigate more fully. In addition, only one hospital site was used, and thus the patient catchment was limited not just by sample size but also by hospital location; it would not be unsurprising if hospitals located in different socioeconomic areas had different demographics for the patient population. But as noted earlier the results obtained serve to provide us with initial insights and directional data for our follow-up research.

### Conclusions

We presented data from an exploratory clinical study designed to establish proof of concept, in an Australian context, of a specific pervasive mobile solution, the Diamond diabetes monitoring device. All patients reported preferring standard care with the technology solution over standard care alone. Further, all clinicians reported that the technology solution greatly assisted their ability to save time and made the monitoring of GDM more efficient and effective and was extremely helpful to assist in systematically monitoring glucose levels, changes, and trends at any time more regularly. Based on these findings, we contend that a pervasive technology solution that is consistent with a value-based care paradigm for chronic disease management is important. Moreover, such a solution should be patient-centric, focused on the two primary user groups (patients and clinicians), and be used in conjunction with standard care protocols. We believe such a solution has the potential to represent a paradigm shift for diabetes care and chronic disease management in general. It is likely that the consequent paradigm shift in the approach to treating chronic diseases such as diabetes will provide the needed impetus to address the rising costs and better means to manage the current state.

In closing, we note that it is essential with all technology solutions, but most especially those in health care, to examine potential risks or negative aspects, if any. Based on the study, no significant risks became apparent, and given the extensive rigor applied in the ethics process, we believe any potential risks were identified during this process and addressed. However, with scale there may be an impact on clinician workload, and this should be investigated in future studies. Thus, establishment of usability, acceptability, and fidelity is clearly a necessary but not sufficient condition for universal adoption of a technology solution in health care contexts.

## Appendix

Multimedia Appendix 1 Design science research methodology.

Multimedia Appendix 2 Questionnaires.

Multimedia Appendix 3 Detailed answers to the posed research questions.

## Abbreviations

DAAD-ATN: German Academic Exchange Service–Australian Technology Network of Universities
GDM: gestational diabetes mellitus
