# Supplementary material for: Achieving Value-Based Care in Chronic Disease Management: Intervention Study
Source: JMIR Diabetes. 2019 May 3;4(2):e10368. doi: 10.2196/10368 (PMC6524451; doi:10.2196/10368)
Supplement: Multimedia Appendix 1 [file diabetes_v4i2e10368_app1.pdf]

**MULTIMEDIA APPENDIX 1**

## Design Science Research Methodology (DSR)

Design science is an important and legitimate research paradigm in information systems (Gregor & Hevner, 2013). Design science research involves constructing a wide range of socio-technical artefacts, such as new software, processes, algorithms or systems intended to improve or solve an identified problem (Myers & Venable, 2014). The design science guidelines originated from information-systems design theory originally proposed by Walls et al. (1992) as "a prescriptive theory which integrates normative and descriptive theories into design paths intended to produce more effective information systems." Eventually, Peffers et al. (2007) expanded design theory into a design science research methodology by incorporating the principles, practices and procedures required to carry out research by applying design science theory. They suggested that design science theory as a methodology, needs to be consistent with prior literature, provide a nominal process model for doing design science research, and provide a mental model for presenting and evaluating design science research (Peffers et al. 2007). Moreover, Hevner and Wickramasinghe (2017) note that in healthcare contexts use of design science research methodology is especially prudent when fine tuning innovative solutions.

Hevner et al. (2004) presented seven guidelines for understanding, executing, and evaluating design science research. Various studies (Nguyen and Wickramasinghe 2017; John et al 2016; Arnott & Pervan 2012; Xu, Wang, Li, & Chau 2007) have used these guidelines for building algorithms and systems. The improved four-cycle model of IS design science research for capturing the dynamic nature of IS artefact design is illustrated in Figure 2 (Drechsler & Hevner 2016). Applying the change and impact cycle to a mobile healthcare application like DiaMonD, the app itself, the mobile device(s), and the patients and/or clinicians that use the app we believed would be prudent and would greatly assist to ensure that the solution was fit for purpose in the Australian healthcare context. The design of DiaMonD using design science research is further discussed in Table A1.

Table A1 **Design Science Research guidelines for DiaMonD**

| Design Science Research guidelines | DiaMonD |
|---|---|
| Guideline 1: Design as an Artefact | DiaMonD – a convenient and innovative mobile solution to support patient and clinical users and enable diabetes self-management and monitoring to ensue. It also has the potential to support a value-based care agenda as it can increase access, has the potential to increase quality of care and more especially timeliness of feedback and does not appear to impact costs of care delivery. |
| Guideline 2: Problem Relevance | To address the need for continuous and superior monitoring and management of GDM patients. To provide in a timely fashion anywhere, anytime key data to facilitate better decision making. To provide an appropriate technology solution that can support self-management of diabetes for both patients and clinicians. |
| Guideline 3: Design Evaluation | Clinicians and potential patient users were included at various points in the design and testing of the solution. In addition, hospital legal representatives were consulted to ensure the solution complied with all government requirements for technology solutions interacting with pregnant women in medical research. This was an iterative process and concluded when legal, clinical and representative patient users were satisfied that the solution was fit for purpose. |

| Design Science Research guidelines | DiaMonD |
|---|---|
| Guideline 4: Research Contributions | In this study, users' perspectives of the mediating role of the solution are explored. |
| Guideline 5: Research Rigor | Theoretical foundations and conceptual models drawn from information systems, chronic disease management protocols, healthcare quality and safety were used to inform the development cycles to evaluate DiaMonD in clinical contexts. |
| Guideline 6: Design as a Search Process | In this project, the design was essential to be correct to meet with ethics requirements in pregnancy type studies and ensure full and complete risk mitigation in such a context. |
| Guideline 7: Communication of Research | Internal communication: Presented the technology and clinically-oriented users through focus groups, simulations exercises, brainstorming meetings, as well as technical and managerial meetings.<br>External communication: Progress and findings are reported in a book chapter and peer review papers submitted to international conferences and professional peer-reviewed journals in relevant disciplines. |
