# Supplementary material for: Achieving Value-Based Care in Chronic Disease Management: Intervention Study
Source: JMIR Diabetes. 2019 May 3;4(2):e10368. doi: 10.2196/10368 (PMC6524451; doi:10.2196/10368)
Supplement: Multimedia Appendix 2 [file diabetes_v4i2e10368_app2.pdf]

APPENDIX 2 Questionnaires

The patient perspective was assessed at four specific stages of the project:

1) A structured questionnaire at the start of the project (PQS);
2) A structured questionnaire at the end of using the standard care (PQES);
3) A structured questionnaire at the end of using the technology solution in conjunction with standard care (PQEST;
4) A structured questionnaire at the end of the project (PQE).

1. Questionnaire – Patient (at the Start of the Project)

Please, complete the questions below as best you can by circling the most correct answer:

1. I use my mobile phone
   a. Never
   b. Only in an emergency
   c. Less than 2 times a week
   d. More than 3 times a day

2. When I use my mobile phone I mainly
   a. Text
   b. Call
   c. Text and call in roughly equal amounts
   d. Call more than text
   e. Text more than call
   f. Other---------------------- (please specify)
   ----

3. I have/use
   a. 1 mobile phone
   b. 2 mobile phones
   c. A mobile phone and IPad (or tablet) and PC

4. The type of mobile phone I use is (please state)

   ----

5. I understand the need to regularly monitor and manage my blood sugar (please circle)

|----------|----------|----------|----------|

1	2	3	4	5
Not at all	Some what unsure	Not sure	Some what understand	Understand

THANK YOU

2. Questionnaire – Patient at the end of Stage 1 of the Project (standard care arm)

Please, complete the questions below as best you can by circling the most correct answer:

1. I understand the need to regularly monitor and manage my blood sugar (please circle)

|----------|----------|----------|----------|

1	2	3	4	5
Not at all	Some what unsure	Not sure	Some what understand	Understand

2. I found the standard care easy to understand (please circle)

|----------|----------|----------|----------|

1	2	3	4	5
Not at all	Some what difficult	Not sure	Some what easy	Very understandable

3. I found the standard care helped me to control my blood sugar(please circle)

|----------|----------|----------|----------|

1	2	3	4	5
Not at all	Not really	Not sure	Some what	Most definitely

4. The following would have been helpful to include: (please list)

THANK YOU

3. Questionnaire – Patient at the end of Stage 1 of the Project (standard care arm + mobile solution)

Please, complete the questions below at best you can by circling the most correct answer:

1. I understand the need to regularly monitor and manage my blood sugar (please circle)

|----------|----------|----------|----------|

1	2	3	4	5
Not at all	Some what unsure	Not sure	Some what understand	Understand

2. I found the mobile solution easy to understand (please circle)

|----------|----------|----------|----------|

1	2	3	4	5
Not at all	Some what difficult	Not sure	Some what easy	Very understandable

3. I found the mobile solution easy to use (please circle)

|----------|----------|----------|----------|

1	2	3	4	5
Not at all	Some what difficult	Not sure	Some what easy	Very easy

4. I found the mobile solution helped me to control my blood sugar(please circle)

|----------|----------|----------|----------|

1	2	3	4	5
Not at all	Not really	Not sure	Some what	Most definitely

5. The following would have been helpful to include: (please list)

----

4. Questionnaire – Patient at the end of the Project

Please, complete the questions below at best you can by circling the most correct answer:

1. I understand the need to regularly monitor and manage my blood sugar (please circle)

```
|----------|----------|----------|----------|
1          2          3          4          5
Not        Some       Not        Some       Understand
at all     what       sure       what
           unsure                understand
```

2. After experiencing standard care and also standard care + the mobile solution I found the key advantages of the mobile solution to be: (please list)

   ----
3. I would recommend the standard care + the mobile solution
   Y/N (please select)
   Why? (please explain)

   ----
4. If I could I would prefer to have the option of standard care + the mobile solution
   Y/N (please select)
   Why? (please list)

   ----
5. Other features I would like to see included with the mobile solution: (please list)

   ----
6. Anything you would like changed with the mobile solution or any other comments: (please list)

Many thanks for participating in our study – have a good day!

Questionnaire – Clinician (at the Start of the Project)

Please, complete the questions below as best you can by circling the most correct answer:

1. I use my mobile phone
   a. Never
   b. Only in an emergency
   c. Less than 2 times a week
   d. More than 3 times a day

2. When I use my mobile phone I mainly
   a. Text
   b. Call
   c. Text and call in roughly equal amounts
   d. Call more than text
   e. Text more than call
   f. Other---------------------- (please specify)
   ----

3. I have/use
   a. 1 mobile phone
   b. 2 mobile phones
   c. A mobile phone and IPad (or tablet) and PC

4. The type of mobile phone I use is (please state)

   ----

5. In general I support my patients to use technology that they want to use (y/n) explain
   ----

THANK YOU

Questionnaire – Clinician at the end of the Project

Please, complete the questions below as best you can by circling the most correct answer:

1. I found the mobile solution easy to work with (please circle)

|----------|----------|----------|----------|

1	2	3	4	5
Not at all	Not really	Not sure	Some what	Definitely

2. I would like to see the following additional features included with the mobile solution: (please list)

----
3. I would suggest the following changes with the mobile solution (please state and explain)

   I believe the mobile solution is better than the standard care approach for assisting patients with GDM to manage and monitor their blood sugar levels?
   Y/N (please circle)
   Why? (please explain)

----
4. I would be happy to recommend using a mobile solution (if available) to monitor and manage blood sugar levels to all my patients who had GDM?
   Y/N (please circle)
   Why? (please explain)

----
5. Any other comments: (please list)

Many thanks for participating in our study – have a good day!

Interview Protocol Clinician

Questions about the trial:

1. Please describe your experience with the technology and standard care arms in the study. What were the key differences, which did you prefer and why? Would you continue to use the solution?

Questions about the technology:

2. Would you recommend this solution to your patients and why?

3. Would you be supportive of your patient using a technology solution you did not recommend why/why not? And if you did not recommend a technology solution do you think your patients would still want to use it why/why not?

4. What are the aspects of a technology solution that must be present vs nice to have vs you don't care for you to recommend the solution to your patients?

Questions about value-based care

5.When you treat patients do you think about access, quality and value issues – please elaborate on how this comes into your thinking?

6.If a solution supports value-based care would you recommend its use to your patients?

Questions about data analytics

7.What types of data would you like to see and how would this help your decision making?

8.Do you think having the data described above would help achieve better treatment results/care and /or would help with population health aspects with regards to GDM? How/Why?
