# Supplementary material for: Achieving Value-Based Care in Chronic Disease Management: Intervention Study
Source: JMIR Diabetes. 2019 May 3;4(2):e10368. doi: 10.2196/10368 (PMC6524451; doi:10.2196/10368)
Supplement: Multimedia Appendix 3 [file diabetes_v4i2e10368_app3.pdf]

Appendix 3 Detailed Answers to the Posed Research Questions

Table A3-1 provides the detailed answers. We believe that the study does highlight the huge potential and benefit for embracing pervasive mobile solutions to facilitate superior care and management in the context of diabetes and other chronic diseases. We also believe that such an approach represents a paradigm shift "a clinician-patient partnership" in the treatment of diabetes which could be helpful to stem current escalating costs and effect better blood glucose control on going.

Table A3-1: Answers to the Posed Research Questions

Question	Answer
How does a mobile solution enable and support the value-based care paradigm in the context of chronic disease management?	As noted earlier, at the essence of this paradigm is that at all times high quality, access and yet minimisation of cost should permeate all healthcare interventions. While no specific economic analysis was conducted in conjunction with this study, it is possible to note that access was superior to the standard care received by patients. Patients received replies to all messages received via the mobile solution at a high level of frequency, and most importantly, if something was deemed of immediate medical importance, an immediate phone call was received. In addition, quality of care was also superior given that it was now possible to provide highly responsive medical interventions as soon as possible. Especially in pregnancy, immediate intervention enables, when required, corrective action to be taken, which has the end result of benefiting both mother and unborn baby. In addition, such quick intervention prevents more complex problems from developing which in turn require more and often more costly intervention. Finally, with respect to cost, patients already had their mobile phones, so they faced no additional costs. The clinicians, in the study, did not identify any significant impact to current costs. In fact they noted that their primary duty is to treat the pregnancy not the GDM per se; and thus anything that assists them to treat GDM more effectively and efficiently is always going to lead to a higher value of care delivery. Moreover, they noted the potential for cost savings by ensuring better glycaemic control, which in turn leads to less complicated births and healthier babies when born. The clinicians also strongly felt that with the technology solution they could provide better care which was their key goal.
What are the benefits and suitability of such a pervasive technology solution to self-care?	The benefits are many including: i) peace of mind for the mother-to-be, ii) convenience for the mother–to-be, especially if she is a working mother, iii) minimisation of travel and waiting times for the mother-to-be which is especially beneficial for more remotely located patients, iv) ability to receive immediate feedback and thus intervene faster if there is a risk or problem. Similarly, benefits for the clinicians include: i) ability to stream line workflow, ii) provide better tailored care to patients, and iii) more effectively manage mother and baby's health.
What are the key barriers and facilitators for the application of a pervasive technology solution to support GDM patient care?	One of the biggest barriers identified is government regulations and policies around pregnancy care which would be needed if the solution were to be embraced on an on-going basis. To offer it as an optional solution is not a problem as long as it complies with all regulations. Key facilitators included clinician and hospital executive support. Patients also intimated that if their clinician did not support the solution they would be reluctant to adopt it while clinicians' clearly stated they would not be so comfortable for their patients to use a technology solution they did not recommend and support because at the end of the day they bear the risk and responsibility. This would also mean that a key facilitator is clinician support and endorsement as well as hospital executive support.
What are the possibilities of applying the tools and	On discussion with clinicians, they believed that collecting the data from all patients would provide deeper insights into GDM – a still less well understood type of diabetes- which would in turn help to inform practice protocols and population health initiatives.

techniques of data science to enable precision healthcare delivery and/or inform public health care initiatives regarding better chronic disease management practices and protocols?	Significant to clinicians was the ability, from analysed data, to give them a clear(er) picture of the current state likely trends and possible impacts to both mother an unborn baby. In this regard, graphical representation was highly welcomed and in fact the graphical view provided by the solution currently was a key factor in why clinicians in particular but also patients found the system so useful.
Are patients influenced and persuaded by their clinician to adopt the solution and is this important in choosing the solution?	Patients noted that if their clinical care team had not used the technology solution, they would not have used the solution even if they found it better than the standard care. Thus, for them it was important that their clinician was also using the technology solution and was supportive of their using the solution. Clinicians also remarked that if their patients used technology solutions that they did not recommend they would not be as comfortable to treat their patients and advise their patients to stop using such solutions.
